# Adjusted association between type 2 immunity and low risk thyroid nodules: a retrospective cohort study

**DOI:** 10.1186/s12902-021-00917-0

**Published:** 2022-01-04

**Authors:** Sanxing Wang, Xia Wang, Xiang Hua, Shichao Jiang, Yong Xie, Hongying Liu

**Affiliations:** 1 Department of Laboratory Medicine, the Second Medical Centre, Chinese PLA General Hospital, Beijing, 100853 China; 2 State Key Laboratory of Medical Molecular Biology and Department of Immunology, Institute of Basic Medical Sciences, Chinese Academy of Medical Sciences, Beijing, 100005 China; 3 National Clinical Research Center for Orthopedics, Sports Medicine & Rehabilitation, Department of Orthopedics, Chinese PLA General Hospital, Beijing, 100853 China; 4 Department of Orthopedics, Shandong Provincial Hospital Affiliated to Shandong First Medical University, Jinan, 250021 China

**Keywords:** Thyroid nodule, Immune responses, Type 2 immunity, Eosinophils

## Abstract

**Background:**

Immune responses, especially type 2 immunity, might be related to the prevalence of thyroid nodules, while the key regulators and potential pathways are remaining largely unknown. In addition, the immune status of individuals could be affected by mixed metabolic background. Herein our aim was to investigate the adjusted association between ultrasound-diagnosed low risk thyroid nodules and immune responses, excluding the interference of metabolic effects on immunity.

**Methods:**

We retrospectively enrolled 1764 subjects who underwent a thorough thyroid ultrasound examination. To eliminate the interference of confounders, we used propensity score matching (PSM) to match age, gender, cigarette smoking and alcohol drinking, parameters that are related with metabolic syndrome (MetS). Then the potential effectors of immune responses involved in the laboratorial assays were evaluated. Binary logistic regression analysis was used to assess the independent predictors of thyroid nodules in a multivariate manner.

**Results:**

The 1172 subjects were remained after PSM, and differences of demographic background between subjects with and without thyroid nodules were eliminated. Metabolic parameters comprising blood pressure, fasting blood glucose, total cholesterol, triglyceride, high-density lipoprotein, low-density lipoprotein and serum uric acid were shown no significant difference between post-PSM subjects with and without thyroid nodules. Among the biochemistry and hematological parameters, white blood cell count and the positive rate of eosinophil percentage were increased in subjects with thyroid nodules than in those without thyroid nodules. In contrast, the positive rate of basophil percentage was lower in subjects with thyroid nodules than in those without thyroid nodules. In addition, the thyroid function test results showed that subjects with thyroid nodules had higher positive rates of antithyroglobulin antibody (TgAb) and antithyroid peroxidase antibody (TPOAb) than subjects without thyroid nodules. The logistic regression analysis indicated that the positive value of TgAb as well as high level of white blood cell count and BMI could serve as independent risk factors of thyroid nodules.

**Conclusions:**

The type 2 immune responses mediated by increased level of eosinophils, along with positive value of TgAb and TPOAb were associated with the presence of thyroid nodules. In addition, the potential role of basophils in protecting against thyroid nodules and the pathogenesis of immune-metabolic status remains to be elucidated.

## Background

Thyroid nodular diseases involve the overgrowth of normal thyroid cells that form a dense lump containing a large volume of the thyroid tissue [1]. Thyroid nodules are a concern across the world, with the prevalence increasing from 19 to 68% in the adult population, as identified by high-resolution ultrasonography [2]. Although the majority of thyroid nodules are noncancerous, a small proportion (7–15%) of them may lead to the development of thyroid cancer [3].

Both branches of the innate and adaptive immune systems interact in the thyroid disorders [4]. With regard to the role of cell-mediated immunity, type 1 immunity promotes activation of mononuclear phagocytes while type 3 immunity recruit neutrophils, which are involved in detection of damage and instigation of tissue repair [5]. While type 2 immunity at tissue barrier sites has broad applicability in immune responses to noxious stimuli, with the immunosurveillance goal of restoring tissue homeostasis, beyond the initial view of antihelminth or atopic reactions [6]. The major cells of type 2 immunity, like eosinophils and basophils, are sources of a wide variety of cytokines, and their functions are increasingly recognized to participate in tissue remodeling and fibrosis [7]. On the other hand, type 2 immunity allows immunoglobulin (Ig) E autoantibodies production against specific antigens, like thyroid peroxidase (TPO) [8]. Those evidences implied that the immune responses, especially type 2 immunity, might be related to the prevalence of thyroid nodules.

However, recent studies have suggested that interference of certain metabolic pathways might be interconnected with dampening distinct cell fates adopted by immune system [9]. In addition, the mixed background of individuals, such as age, gender, cigarette smoking and alcohol drinking, could affect the immune status of individuals due to metabolic regulation [9, 10]. Moreover, metabolic syndrome (MetS) was reportedly associated with the presence of thyroid nodules [11]. Besides, several well-known tumor markers, like carcinoembryonic antigen (CEA), carbohydrate antigen 19–9 (CA19–9) and calcitonin levels [12], have been proposed to be prthyroid noduleognostic factor in metastatic medullary thyroid carcinoma [13]; however, its role between tumor immunity and low risk thyroid nodules needs further elucidation. Thus, the objective of the present study was to investigate the adjusted association between thyroid nodules and immune responses, excluding the mixed confounders, and reveal the underlying effect and potential mechanism of immune systems on the formation of low risk thyroid nodules.

## Methods

### Study participants

We originally involved 1924 participants aged between 18 and 90 years who routinely underwent health checkups at the Chinese PLA General Hospital (CPLAGH, Beijing, China) from August 2017 to November 2020. All the subjects were resident population in Beijing, a universal iodised salt city, and were considered as iodine adequate. Professional physicians were responsible for the physical examination of the participants as well as for recording demographic and anthropometric data, past medical history (e.g., diseases, surgeries) and past or current habits [cigarette smoking (yes/no), alcohol drinking (yes/no)]. Then, subjects with history of thyroid diseases, thyroid interventions, debilitating diseases, liver and kidney dysfunction, hematological and connective tissue diseases, malignant tumors, as well as long-term use of hormones and immunosuppressive drugs (continuous use of drugs for > 6 months) were excluded.

### Ultrasound measurements

**Fig. 1:**
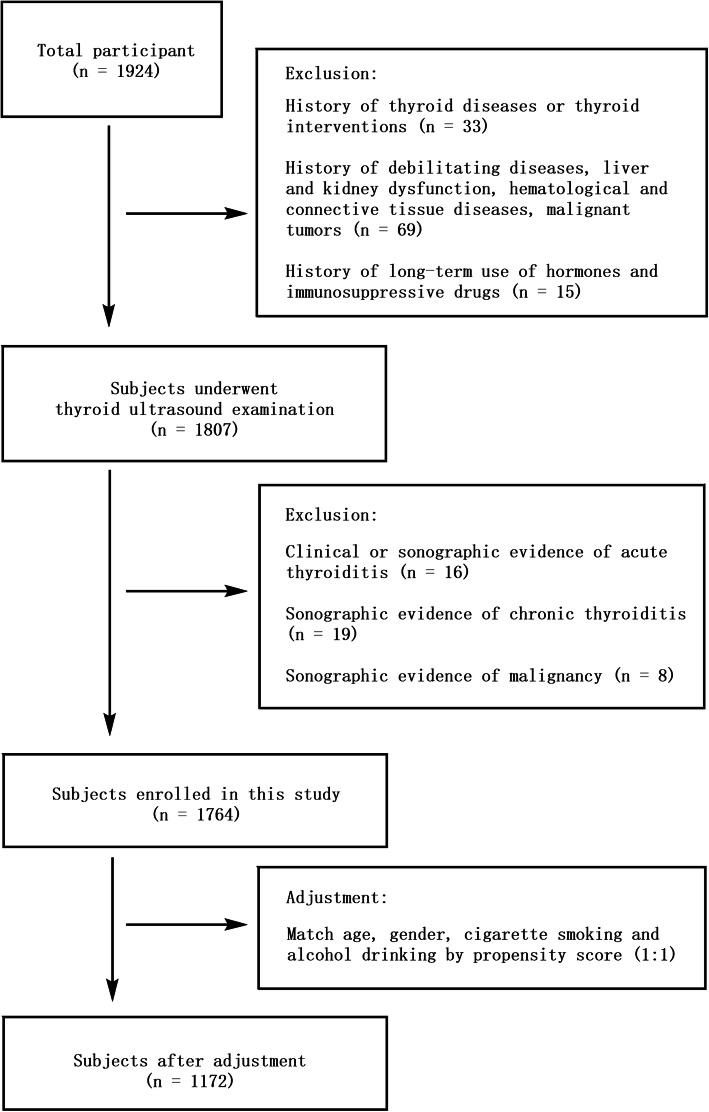
Flowchart of participants’ inclusion and exclusion

The 1807 subjects who were remained after exclusion, underwent a thorough thyroid ultrasound examination, which was performed with high-resolution sonographic devices (7–9 MHz linear probes) using conventional B-mode scanning. A diameter of ≥5 mm was used to determine the presence of thyroid nodules by a specialized ultrasound radiologist, in addition, the diagnosis of low risk thyroid nodule has been reviewed and confirmed by the other two endocrinologists on the basis of clinical or sonographic evidences (purely cystic nodules, spongiform or partially cystic nodules, isoechoic or hyperechoic solid nodule without microcalcification, irregular margin or extrathyroidal extension, or taller than wide shape) [14], the patients with suspicious sonographic features of malignancy [at least 1 of the following: hypoechogenicity, irregular margins, a more tall (anteroposterior) than wide (transverse) shape of the nodule, intranodular vascular spots, bmicrocalcifications; largest diameter of 10 mm or more] were excluded [15] and recommended to reassessment by fine-needle aspiration cytology (the biopsy results have not been followed up). All the subjects were divided into two groups (With or Without low risk thyroid nodule) after excluding those with painful thyroid swelling, and sonographic features of acute or chronic thyroiditis [16], we finally enrolled 1764 subjects for subsequent analysis (Fig. 1).

### Anthropometric and laboratory analyses

An information collection table was established. Body mass index (BMI) was calculated as weight in kilograms per square of height in meters (kg/m^2^). Fat mass, total body fat percentage (%TBF), muscle mass, and basal metabolic rate were assessed via foot-to-foot bioelectrical impedance analysis with a body composition analyzer (X-Scan plus II, ACCUNIQ, Daejeon, Korea). Venous blood was collected on an empty stomach and evaluated using an automatic biochemical analyzer; data pertaining to routine blood indices, biochemistry and hematology analysis, thyroid function and serum tumor markers were recorded. All procedures performed in studies involving human participants were in accordance with the ethical standards of the institutional and/or national research committee and approved by the Scientific and Ethics Committee of the CPLAGH.

### Statistical analysis

Values are presented as percentages for categorical variables, and continuous variables are reported as mean ± standard deviation (SD) or median (interquartile range). Statistical differences in means of continuous variables (normally distributed) or proportions between individuals with or without thyroid nodules were assessed with t-test and chi-squared tests, while the non-parametric tests (Wilcoxon/Kruskal-Wallis H test) were used for determining statistical differences in medians of continuous variables which are not normally distributed. R programming language-based statistics package was used for propensity score matching (PSM) to eliminate the baseline differences of matching variables, including age, gender, cigarette smoking and alcohol drinking (matching algorithm: nearest-neighbour matching; estimation algorithm: logistic regression; matching order: random; caliper: 0.1). The 1:1 matched subjects (*n* = 1172) were extracted from the original enrolled subjects (*n* = 1764). The statistical significances of those adjusted variables were calculated in a multiple comparison manner firstly. The main clinical parameters that were clarified non-normal distribution, such as eosinophil percentage, basophils percentage, values of TgAb and TPOAb, were re-evaluated to binary variables (positive/negative) according to their medical reference range. The statistically differences of the positive rates between groups were assessed by chi-squared tests, as to representing the clinical significance. Then, binary logistic regression analysis was used to assess the independent predictors of thyroid nodules in a multivariate manner [dependent variable (1 = subjects with thyroid nodules, 0 = subjects without thyroid nodules), which took account all variables of differences between-group by calculating odds ratio (OR) and 95% confidence interval (95% CI). Statistical analysis was performed with IBM SPSS Statistics, Version 26 (IBM Corporation, Armonk, NY, USA). Two-tailed *P* < 0.05* or *P* < 0.01** indicated statistical significance.

## Results

### Original and adjusted demographic and anthropometric parameters of subjects with and without thyroid nodules

We included 1764 subjects underwent a thorough thyroid ultrasound examination for analysis. Among them, 897 subjects were diagnosed low risk thyroid nodules versus (vs) 867 subjects revealed non thyroid nodule existed. Individuals with thyroid nodules were older (51.39 ± 10.06 years vs 46.34 ± 9.99 years, *P* < 0.01), more frequently female gender (47.27% vs 32.76%, *P* < 0.01), accompanied by cigarette smoking (28.26% vs 21.96%, *P* < 0.01) and alcohol drinking (26.30% vs 22.28%, *P* < 0.01). In addition, the anthropometric parameters related to metabolic status including BMI and %TBF were higher in subjects with thyroid nodules than subjects without thyroid nodules. In contrast, basal metabolic rate [1267.6 ± 186.9 kJ/(m^2^·h) vs 1326.95 ± 182.02 kJ/(m^2^·h), *P* < 0.01] and muscle mass (46.65 ± 9.18 vs 48.08 ± 8.68, *P* < 0.01) were lower in those with thyroid nodules. After the PSM for demographic background that age, gender, cigarette smoking and alcohol drinking, 1172 subjects were remained (586 with thyroid nodules vs 586 without thyroid nodules, Fig. 1), and the original between-group difference of anthropometric parameters including basal metabolic rate and systolic blood pressure were eliminated. While the adjusted BMI (25.24 ± 3.59 kg/m^2^ vs 24.53 ± 3.31 kg/m^2^, *P* < 0.01) and %TBF (25.76 ± 5.12 vs 25.17 ± 4.96, *P* < 0.05) of subjects with thyroid nodules were shown higher level than those of subjects without thyroid nodules (Tables 1 and 2).

**Table 1:** Original demographic and anthropometric parameters of subjects with and without thyroid nodules

Variables	With thyroid nodules (*N* = 897)	Without thyroid nodules (*N* = 867)	*P* value
Age (year, X ± s)	51.39 ± 10.06	46.34 ± 9.99	< 0.01**
% of women	47.27	32.76	< 0.01**
Cigarette smoking (%)	28.26	21.96	< 0.01**
Alcohol drinking (%)	26.30	22.28	< 0.01**
Body mass index (kg/m^2^, X ± s)	24.74 ± 3.51	24.33 ± 3.48	< 0.01**
%TBF	26.75 ± 5.36	24.94 ± 5.09	< 0.01**
Heart rate (/min)	70.08 ± 8.55	69.78 ± 8.83	0.46
Basal metabolic rate [kJ/(m^2^·h)]	1267.56 ± 186.85	1326.95 ± 182.02	< 0.01**
Systolic blood pressure (mmHg)	117.17 ± 18.65	114.92 ± 16.59	< 0.01**
Diastolic blood pressure (mmHg)	79.78 ± 10.15	79.71 ± 9.82	0.88

Values represent mean ± standard deviation (X ± s), unless stated otherwise

*%TBF* total body fat percentage

*P < 0.05, **P < 0.01

**Table 2:** Adjusted demographic and anthropometric parameters based on PSM

Variables	With thyroid nodules (*N* = 586)	Without thyroid nodules (*N* = 586)	*P* value
**Age (year, X ± s)**	49.42 ± 8.97	49.09 ± 9.15	0.95
**% of women**	63.80	64.00	0.89
**Cigarette smoking (%)**	14.80	15.30	0.70
**Alcohol drinking (%)**	29.30	28.90	0.81
Body mass index (kg/m^2^, X ± s)	25.24 ± 3.59	24.53 ± 3.31	< 0.01**
%TBF	25.76 ± 5.12	25.17 ± 4.96	< 0.05*
Heart rate (/min)	69.64 ± 8.30	69.47 ± 8.90	0.41
Basal metabolic rate [kJ/(m^2^·h)]	1335.85 ± 183.46	1316.29 ± 168.23	0.06
Systolic blood pressure (mmHg)	118.04 ± 17.60	116.38 ± 17.03	0.46
Diastolic blood pressure (mmHg)	79.85 ± 9.84	79.46 ± 9.97	0.51

Matching variables included age, gender, cigarette smoking and alcohol drinking

Values represent mean ± standard deviation (X ± s), unless stated otherwise

*%TBF* total body fat percentage

*P < 0.05, **P < 0.01

### Biochemistry and hematological parameters of subjects with and without thyroid nodules

Based on PSM, we found that the metabolic parameters including fasting blood glucose, total cholesterol, triglyceride, high-density lipoprotein, low-density lipoprotein, alkaline phosphatase were shown no difference between subjects with and without thyroid nodules. Besides, there were no marked between-group differences in the other hepatic function index and electrolyte concentration like alanine aminotransferase, aspartate transaminase, total bilirubin, direct bilirubin, uric acid and inorganic phosphate. However, the serum calcium (2.34 ± 0.08 vs 2.33 ± 0.07, *P* < 0.05) of subjects with thyroid nodules was higher than those of subjects without thyroid nodules. The mean value of biochemistry and hematological parameters were within normal range either in the subjects with or without thyroid nodules (Table 3).

**Table 3:** Biochemistry and hematological parameters of subjects with and without thyroid nodules (based on PSM)

Variables	With thyroid nodules (*N* = 586)	Without thyroid nodules (*N* = 586)	*P* value
Fasting blood glucose (mmol/L)	5.77 ± 1.15	5.75 ± 1.25	0.90
Total cholesterol (mmol/L)	4.80 ± 0.91	4.75 ± 0.93	0.27
Triglyceride (mmol/L)	1.66 ± 0.98	1.54 ± 1.06	0.65
High-density lipoprotein (mmol/L)	1.22 ± 0.31	1.25 ± 0.32	0.38
Low-density lipoprotein (mmol/L)	3.13 ± 0.85	3.09 ± 0.83	0.91
Alkaline phosphatase (U/L)	68.12 ± 18.26	66.25 ± 19.31	0.13
Alanine aminotransferase (U/L)	25.53 ± 16.84	23.71 ± 15.63	0.13
Aspartate transaminase (U/L)	21.17 ± 8.45	20.40 ± 8.12	0.58
Total bilirubin (μmol/L)	11.92 ± 5.45	12.48 ± 6.68	0.68
Direct bilirubin (μmol/L)	4.16 ± 1.60	4.26 ± 1.54	0.90
Serum calcium (mmol/L)	2.34 ± 0.08	2.33 ± 0.07	< 0.05*
Serum uric acid (mmol/L)	346.04 ± 85.65	343.90 ± 88.96	0.35
Inorganic phosphate (mmol/L)	1.13 ± 0.15	1.13 ± 0.14	0.99

Matching variables included age, gender, cigarette smoking and alcohol drinking. Values represent mean ± standard deviation (X ± s)

*P < 0.05, **P < 0.01

### Blood indices of subjects with and without thyroid nodules

With regard to routine blood indices, white blood cell count (6.31 ± 1.65 10^9^/L vs 6.01 ± 1.40 10^9^/L, *P* < 0.01) and platelet count (228.41 ± 52.41 10^9^/L vs 221.58 ± 48.12 10^9^/L, *P* < 0.05) were higher in the subjects with thyroid nodules than in subjects without thyroid nodules, while the mean values of both groups were within normal range. In addition, the positive rate of eosinophil percentage (5.1% vs 3.5%, *P* < 0.05) was higher in subjects with thyroid nodules than in those without thyroid nodules. In contrast, the positive rate of basophils percentage (2.3% vs 4%, *P* < 0.01) was lower in those without thyroid nodules (Table 4).

**Table 4:** Blood indices of subjects with and without thyroid nodules (based on PSM)

Variables	With thyroid nodules (*N* = 586)	Without thyroid nodules (*N* = 586)	*P* value
White blood cell count (10^9^/L)	6.31 ± 1.65	6.01 ± 1.40	< 0.01**
Erythrocyte count (10^9^/L)	4.93 ± 0.45	4.89 ± 0.46	0.80
Platelet count (10^9^/L)	228.41 ± 52.41	221.58 ± 48.12	< 0.05*
Neutrophil(%)	0.59 ± 0.07	0.59 ± 0.07	0.95
Monocytes (%)	0.06 ± 0.01	0.06 ± 0.01	0.81
Lymphocytes (%)	0.31 ± 0.06	0.32 ± 0.06	0.83
Eosinophils (%) (positive rate, >0.01%)	5.1	3.5	< 0.05*
Basophils (%) (positive rate, >0.05%)	2.3	4	< 0.01**
Hematocrit (L/L)	0.43 ± 0.03	0.43 ± 0.03	0.75
Hemoglobin (g/L)	149.60 ± 15.09	149.03 ± 14.48	0.28
Mean corpuscular hemoglobin concentration (g/L)	343.67 ± 10.24	343.79 ± 9.43	0.14

Matching variables included age, gender, cigarette smoking and alcohol drinking. Values represent mean ± standard deviation (X ± s), the eosinophils percentage and basophils percentage were clarified non-normal distribution, shown as positive rate in terms of the medical reference range

*P < 0.05, **P < 0.01

### Comparison of thyroid-function and serum tumor markers of subjects with and without thyroid nodules

Thyroid function test results showed that the positive rates of antithyroglobulin antibody (TgAb, 4.6% vs 2.3%, *P* < 0.05) and antithyroid peroxidase antibody (TPOAb, 4.4% vs 2.8%, *P* < 0.05) were higher in those with than in those without thyroid nodules. While there were no statistically significant differences between the two groups regarding the serum levels of thyroid-stimulating hormone (TSH), free T3 and free T4 (Table 5). Besides, we didn’t detect any difference for serum tumor markers compromised alpha-fetoprotein (AFP), carcinoembryonic antigen (CEA), neuron-specific enolase (NSE), carbohydrate antigen 19–9 (CA19–9), carbohydrate antigen 12–5 (CA12–5) and carbohydrate antigen 15–3 (CA15–3) in between subjects with and without thyroid nodules (Table 6).

**Table 5:** Thyroid function test results of subjects with and without thyroid nodules (based on PSM)

Variables	With thyroid nodules (*N* = 586)	Without thyroid nodules (*N* = 586)	*P* value
TgAb (positive rate, ≥115 IU/L)	4.6	2.3	< 0.05*
TPOAb (positive rate, ≥34 IU/L)	4.4	2.8	< 0.05*
TSH (median, IQR mU/L)	1.95 (1.36)	1.96 (1.44)	0.27
Free T3 (pmol/L)	5.24 ± 0.88	5.20 ± 0.77	0.45
Free T4 (pmol/L)	16.56 ± 2.52	16.56 ± 2.13	0.26

Matching variables included age, gender, cigarette smoking and alcohol drinking

Values represent mean ± standard deviation (X ± s), the values of TgAb, TPOAb and TSH were clarified non-normal distribution, shown as positive rate in terms of the medical reference range or IQR (interquartile range)

*TgAb* thyroglobulin antibody, *TPOAb* antithyroid peroxidase antibody, *TSH* thyroid-stimulating hormone

*P < 0.05, **P < 0.01

**Table 6:** Serum tumor markers of subjects with and without thyroid nodules (based on PSM)

Variables	With thyroid nodules (*N* = 586)	Without thyroid nodules (*N* = 586)	*P* value
AFP (μg/L)	3.64 ± 1.92	3.61 ± 1.80	0.09
CEA (μg/L)	2.08 ± 1.28	2.12 ± 1.55	0.50
NSE (ng/mL)	11.32 ± 2.06	11.42 ± 2.10	0.59
CA19–9 (μ/L)	10.88 ± 9.21	10.53 ± 8.87	0.44
CA12–5 (μ/mL)	11.54 ± 8.10	11.09 ± 7.85	0.11
CA15–3 (μ/mL)	9.33 ± 4.37	9.58 ± 5.26	0.36

Matching variables included age, gender, cigarette smoking and alcohol drinking

Values represent mean ± standard deviation (X ± s)

*AFP* alpha-fetoprotein, *CEA* carcinoembryonic antigen, *NSE* neuron-specific enolase, *CA19–9* carbohydrate antigen 19–9, *CA12–5* cancer antigen 125, *CA15–3* cancer antigen 153

*P < 0.05, **P < 0.01

### Assessment of predictors of the presence of thyroid nodules

Furthermore, we performed binary logistic regression analyses to assess the independent predictors of thyroid nodules in a multivariate manner [dependent variable (1 = subjects with thyroid nodules, 0 = subjects without thyroid nodules), which took account all variables of differences between-group and demographic background including age, gender, cigarette smoking and alcohol drinking by calculating OR and 95% CI. The results demonstrated that positive rate of TgAb [OR 2.023, 95% CI 1.121–3.652, *P* < 0.05], white blood cell count (OR 1.132, 95% CI 1.037–1.235, *P* < 0.01) and BMI (OR 1.116, 95% CI 1.047–1.190, *P* < 0.01) were associated with the presence of thyroid nodules. In contrast, the subjects with high positive rate of basophils percentage (OR 0.577, 95% CI 0.347–0.958, *P* < 0.01) had decreased risk of developing thyroid nodules. No other significant association was detected (Table 7).

**Table 7:** Independent predictors of thyroid nodules identified by binary logistic regression analyses (based on PSM)

Variables	Binary logistic regression analysis		
	Odds ratio (OR)	95% confidence interval	*P* value
TgAb (positive rate, ≥115 IU/L)	2.023	1.121–3.652	< 0.05*
TPOAb (positive rate, ≥34 IU/L)	1.175	0.670–2.058	0.57
Serum calcium (mmol/L)	4.650	0.995–21.731	0.051
White blood cell count (10^9^/L)	1.132	1.037–1.235	< 0.01**
Basophils (%) (positive rate, >0.05%)	0.577	0.347–0.958	< 0.05*
Eosinophils (%) (positive rate, >0.01%)	1.405	0.909–2.137	0.12
Platelet count (10^9^/L)	1.001	0.999–1.004	0.39
Body mass index (kg/m^2^, X ± s)	1.116	1.047–1.190	< 0.01**
%TBF	0.956	0.913–1.001	0.054

Matching variables included age, gender, cigarette smoking and alcohol drinking

Values represent mean ± standard deviation (X ± s), the values of TgAb, TPOAb, basophils percentage and eosinophils percentage were clarified non-normal distribution, shown as positive rate in terms of the medical reference

*TgAb* thyroglobulin antibody, *%TBF* total body fat percentage

*P < 0.05, **P < 0.01

## Discussion

In the present study, 1172 individuals who underwent thyroid ultrasound examination were finally enrolled in the analysis after the PSM for the mixed background. We found that the increased number of white blood cells, along with upregulation in positive rate of basophils percentage, were associated with the presence of low risk thyroid nodules. Several studies have reported that upregulation of white blood cell count and basophils percentage can represent chronic inflammation, which play roles in the nodular enlargement of thyroid [17–19]. Blood cells comprised neutrophils, eosinophils, basophils, lymphocytes and monocytes, most of them increase significantly in case of infection, while some of these parameters will increase in noninfectious diseases with low-grade inflammation. Through the comprehensive analysis conducted in present study, we identified that upregulated white blood cell count could be used as an independent predictor of thyroid nodules. Furthermore, we demonstrated that the increased positive rate of eosinophils percentage, along with downregulated positive rate of basophils percentage in subjects with thyroid nodules.

In contrast to the involvement in allergic inflammatory diseases and helminth parasite infections [20], eosinophils, multifunctional leucocytes were produced as over-immune responses to tissue damage caused by autoimmune and endocrine disorders, such as Riedel’s thyroiditis and pulmonary fibrosis, which were frequently associated with tissue remodeling and fibrosis [21]. A large number of eosinophils secrete interleukin (IL)-6, which can be recruited to specific niches and contribute to the innate mucosal immunity [22]. The cationic proteins were induced by eosinophils, then mediated the proliferation and trans-differentiation from fibroblast to myofibroblasts, which were involved in local fibrogenesis via collagen gel contraction, and expression of various matrix metalloproteinases [23, 24]. Studies suggested that IL-5 promotes the activation and survival of mature eosinophils in the periphery and stimulates autophagy in eosinophils that regulates the severity of eosinophilic inflammation [25]. A humanized monoclonal anti-IL-5 has been evaluated for treatment of hypereosinophilic syndromes that affects fibrogenesis [26]. A series of studies demonstrated that IL-5 modifies eosinophil regulation of adipose tissue and glucose homeostasis by sustaining alternatively activated M2 macrophages [22]. While the production of IL-5 and accumulation of eosinophils were promoted by innate lymphoid type 2 cell (ILC2) [27]. Although we have adjusted the effects of mixed background regarding the MetS, our analysis demonstrated that BMI and %TBF were shown higher level in subjects with thyroid nodules than those of subjects without thyroid nodules. It has been reported that augmentation or interference of certain metabolic pathways might be useful in clinical intervention of immune disorders [28]. The cell fates of macrophages and T cells were largely adopted by their metabolic actions [9]. Though the coordination between leukocyte activity and metabolic status has not been fully elucidated, immune-metabolic cross-talk shown important role in chronic inflammation and fibrogenesis [28]. Besides, evidences have suggested that eosinophil migration to thyroids occurs as a consequence of Interferon (IFN)-c deficiency, but these cells have no apparent pathogenic role for autoimmune thyroiditis but tissues enlargement [26]. Whereas the influx of eosinophils into gland tissues depends on the local expression of eotaxins, the underling mechanisms that affect functioning of eosinophils in formation of thyroid nodules remain to be delineated [29].

In the present study, the positive rate of basophils percentage was shown an unexpected downregulation in subjects with low risk thyroid nodules. Accounting for around 1% of white blood cells, basophils are important in promoting type 2 immune responses via their production of effector molecules. IL-6 can be activated by recognition of parasite-derived antigens via IgE bound to its high-affinity receptor FcεRI on the cell surface of basophils [30]. Evidence has been found that basophil-derived IL-6 is engaged in immune memory response through stimulating Th17 cell differentiation [31]. In addition, basophil was known as “innate IL-4 producing cell” and involved in a complex cross talk in the immune regulation. Therefore, the downregulation of basophils may result in low levels of both IL-4 and IL-6, which might be associated with the disruption of normal thyroid tissue remodeling. Additionally, our post-PSM analysis demonstrated that the serum calcium and platelet count in subjects with thyroid nodules was higher than in those without thyroid nodules. Although it has been reported that low levels of serum calcium may predict the existence of nonmedullary thyroid carcinoma in primary hyperparathyroidism patients with thyroid nodules [32], the association between serum calcium and thyroid nodules still remains unclear. Increase of mean platelet volume (MPV) has been described in various inflammatory conditions [33]. Moreover, platelet count was proved that positively correlated with serum levels of C-reactive protein (CRP) in colorectal cancer (CRC) [34]. Platelets contain microparticles that were involved in trafficking inflammatory molecules (e.g., CD40L and IL-1) into immune cells, which can shape both innate and adaptive immunity. In addition, platelets express several functional chemokine receptors, such as CCR3, and CXCR4, which are involved in the critical network facilitating immune cell and surveillance [35].

The positive rates of TgAb and TPOAb were significantly upregulated in those with thyroid nodules. Immunoglobulin (Ig) E autoantibodies against TPO have been demonstrated in chronic spontaneous urticaria (CSU) patients in higher frequency than healthy subjects [8]. IgE against TPO plays a pathogenic role in inducing effector cell activation and skin exacerbation in some patients with CSU. Type 2 immunity consists of ILC2s and TH2 cells producing IL-5 and IL-13, which induce IgE antibody [5]. While the presence of TgAb and TPOAb might be an indicator of autoimmune disorder, which may increase the risk of inflammation and tissue damage of the thyroid [36–38]. Refers to the tumor makers, we didn’t obtain any direct evidences that indicate the connection between potential tumor immune response and thyroid nodules. Therefore, the formation of low risk thyroid nodules could be over-immune responses of thyroid tissues to the inflammatory irritation [39]. According to our finding in logistic regression analysis, routine thyroid ultrasound examination is a recommendation for the individuals with high level of white blood cell count and BMI, especially accompanied by positive of eosinophil percentage, TPOAb and TgAb. In addition, individuals with positive value of basophils percentage might have a lower susceptibility to thyroid nodules.

## Limitation

In the study, we have excluded the main confounders that could affect the immune status of individuals due to metabolic regulation, such as age, gender, cigarette smoking and alcohol drinking, all of which were demonstrated to be associated with the presence of thyroid nodules. However, after the PSM, the adjusted BMI and %TBF still showed slight differences, which might be confounders affect metabolic-immune interaction. Besides, the patients with suspicious sonographic features of malignancy were excluded and recommended to reassessment by fine-needle aspiration cytology, while their biopsy results have not been followed up in the present study. The cytologically low risk thyroid nodules can be classified into low risk as well. Further study is needed to eliminate all the impact of metabolic confounders and include the cytologically low risk thyroid nodules.

## Conclusion

The type 2 immune responses mediated by increased percentage of eosinophil, along with positive values of TgAb and TPOAb were associated with the presence of thyroid nodules. The positive value of TgAb as well as high level of white blood cell count and BMI could serve as independent risk factors of thyroid nodules. While the potential role of basophils in protecting against thyroid nodules and the pathogenesis of immune-metabolic status remains to be elucidated.

## Data Availability

The datasets used and/or analyzed during the current study are available from the corresponding author on reasonable request.

## Abbreviations

%TBF: Total body fat percentage
AFP: Alpha-fetoprotein
BMI: Body mass index
CA12–5: Carbohydrate antigen 12–5
CA15–3: Carbohydrate antigen 15–3
CA19–9: Carbohydrate antigen 19–9
CEA: Carcinoembryonic antigen
CRC: Colorectal cancer
CRP: C-reactive protein
CSU: Chronic spontaneous urticaria
IFN: Interferon
Ig: Immunoglobulin
IL: Interleukin
ILC2: Innate lymphoid type 2 cell
IQR: Interquartile range
MetS: Metabolic syndrome
MPV: Mean platelet volume
NSE: Neuron-specific enolase
PSM: Propensity score matching
TgAb: Antithyroglobulin antibody
TPO: Thyroid peroxidase
TPOAb: Antithyroid peroxidase antibody
TSH: Thyroid-stimulating hormone
SD: Standard deviation

## References

[1] Migdal AL, Sternberg SB, Oshin A, Aronson MD, Hennessey JV (2016). Building a quality management system for a thyroid nodule clinic. Thyroid.

[2] Wong R, Farrell SG, Grossmann M (2018). Thyroid nodules: diagnosis and management. Med J Aust.

[3] Burman KD, Wartofsky L (2015). Clinical practice. Thyroid nodules. N Engl J Med.

[4] Ward LS (2014). Immune response in thyroid cancer: widening the boundaries. Scientifica.

[5] Annunziato F, Romagnani C, Romagnani S (2015). The 3 major types of innate and adaptive cell-mediated effector immunity. J Allergy Clin Immunol.

[6] Lloyd CM, Snelgrove RJ (2018). Type 2 immunity: expanding our view. Sci Immunol.

[7] Gieseck RL, Wilson MS, Wynn TA (2018). Type 2 immunity in tissue repair and fibrosis. Nat Rev Immunol.

[8] Sánchez J, Sánchez A, Cardona R (2019). Causal relationship between anti-TPO IgE and chronic urticaria by in vitro and in vivo tests. Allergy, Asthma Immunol Res.

[9] Ganeshan K, Chawla A (2014). Metabolic regulation of immune responses. Annu Rev Immunol.

[10] Mehran L, Amouzegar A, Azizi F (2019). Thyroid disease and the metabolic syndrome. Curr Opin Endocrinol Diabetes Obes.

[11] Iwen KA, Oelkrug R, Kalscheuer H, Brabant G (2018). Metabolic syndrome in thyroid disease. Front Horm Res.

[12] Milman S, Arnold JL, Price M, Negassa A, Surks MI, Fleischer N, Whitney KD (2015). Medullary thyroid cancer that stains negative for CA 19-9 has decreased metastatic potential. Endocr Pract.

[13] Alencar R, Kendler DB, Andrade F, Nava C, Bulzico D, Cordeiro de Noronha Pessoa C, Corbo R, Vaisman F (2019). CA19-9 as a predictor of worse clinical outcome in medullary thyroid carcinoma. Eur Thyroid J.

[14] Haugen BR, Alexander EK, Bible KC, Doherty GM, Mandel SJ, Nikiforov YE, Pacini F, Randolph GW, Sawka AM, Schlumberger M (2016). 2015 American thyroid association management guidelines for adult patients with thyroid nodules and differentiated thyroid cancer: the American thyroid association guidelines task force on thyroid nodules and differentiated thyroid cancer. Thyroid.

[15] Gharib H, Papini E, Paschke R, Duick DS, Valcavi R, Hegedüs L, Vitti P (2010). American association of clinical endocrinologists, associazione medici endocrinologi, and European thyroid association medical guidelines for clinical practice for the diagnosis and management of thyroid nodules. Endocr Pract.

[16] Durante C, Costante G, Lucisano G, Bruno R, Meringolo D, Paciaroni A, Puxeddu E, Torlontano M, Tumino S, Attard M (2015). The natural history of benign thyroid nodules. Jama.

[17] Penninx BW, Pahor M, Cesari M, Corsi AM, Woodman RC, Bandinelli S, Guralnik JM, Ferrucci L (2004). Anemia is associated with disability and decreased physical performance and muscle strength in the elderly. J Am Geriatr Soc.

[18] Ding L, Xu Y, Wang LM, Jiang Y, Zhang M, Li YC, Lu JL, Xu M, Wang TG, Li M (2016). Smoking and its relation to metabolic status among Chinese adults: analysis of a nationwide survey. Biomed Environ Sci.

[19] Schrieks IC, Heil AL, Hendriks HF, Mukamal KJ, Beulens JW (2015). The effect of alcohol consumption on insulin sensitivity and glycemic status: a systematic review and meta-analysis of intervention studies. Diabetes Care.

[20] Weller PF, Spencer LA (2017). Functions of tissue-resident eosinophils. Nat Rev Immunol.

[21] Sakkal S, Miller S, Apostolopoulos V, Nurgali K (2016). Eosinophils in cancer: favourable or unfavourable?. Curr Med Chem.

[22] Klion AD, Ackerman SJ, Bochner BS (2020). Contributions of eosinophils to human health and disease. Annu Rev Pathol.

[23] Travers J, Rothenberg ME (2015). Eosinophils in mucosal immune responses. Mucosal Immunol.

[24] Wen T, Rothenberg ME. The regulatory function of eosinophils. Microbiol Spectr. 2016;4(5). 10.1128/microbiolspec.MCHD-0020-2015. 10.1128/microbiolspec.MCHD-0020-2015 PMC5088784 27780017

[25] Germic N, Frangez Z, Yousefi S, Simon HU (2019). Regulation of the innate immune system by autophagy: neutrophils, eosinophils, mast cells, NK cells. Cell Death Differ.

[26] Fang Y, Chen K, Jackson DA, Sharp GC, Braley-Mullen H (2010). Eosinophils infiltrate thyroids, but have no apparent role in induction or resolution of experimental autoimmune thyroiditis in interferon-gamma(−/−) mice. Immunology.

[27] McLaughlin T, Ackerman SE, Shen L, Engleman E (2017). Role of innate and adaptive immunity in obesity-associated metabolic disease. J Clin Invest.

[28] Andersen CJ, Murphy KE, Fernandez ML (2016). Impact of obesity and metabolic syndrome on immunity. Adv Nutr.

[29] Yang BG, Seoh JY, Jang MH (2017). Regulatory eosinophils in inflammation and metabolic disorders. Immune Netw.

[30] Eberle JU, Voehringer D (2016). Role of basophils in protective immunity to parasitic infections. Semin Immunopathol.

[31] Chirumbolo S, Bjørklund G, Sboarina A, Vella A (2018). The role of basophils as innate immune regulatory cells in allergy and immunotherapy. Hum Vaccin Immunotherapeutics.

[32] Xue Y, Ye ZQ, Zhou HW, Shi BM, Yi XH, Zhang KQ (2016). Serum calcium and risk of nonmedullary thyroid cancer in patients with primary hyperparathyroidism. Med Sci Monit.

[33] Sit M, Aktas G, Ozer B, Kocak MZ, Erkus E, Erkol H, Yaman S, Savli H (2019). Mean platelet volume: an overlooked herald of malignant thyroid nodules. Acta Clin Croat.

[34] Väyrynen JP, Väyrynen SA, Sirniö P, Minkkinen I, Klintrup K, Karhu T, Mäkelä J, Herzig KH, Karttunen TJ, Tuomisto A (2019). Platelet count, aspirin use, and characteristics of host inflammatory responses in colorectal cancer. J Transl Med.

[35] Li C, Li J, Ni H (2020). Crosstalk between platelets and microbial pathogens. Front Immunol.

[36] Wong SL, Grodski S, Yeung MJ, Serpell JW (2015). Anti-thyroid antibodies as a predictor of thyroid cancer. ANZ J Surg.

[37] Gerard AC, Boucquey M, van den Hove MF, Colin IM (2006). Expression of TPO and ThOXs in human thyrocytes is downregulated by IL-1alpha/IFN-gamma, an effect partially mediated by nitric oxide. Am J Phys Endocrinol Metab.

[38] Xu W, Huo L, Chen Z, Huang Y, Jin X, Deng J, Zhu S, Yu Y (2017). The relationship of TPOAb and TGAb with risk of thyroid nodules: a large epidemiological study. Int J Environ Res Public Health.

[39] Marzullo P, Minocci A, Mele C, Fessehatsion R, Tagliaferri M, Pagano L, Scacchi M, Aimaretti G, Sartorio A (2018). The relationship between resting energy expenditure and thyroid hormones in response to short-term weight loss in severe obesity. PLoS One.

